# Immunohistochemical study to detect glucagon and insulin hormones in pancreas of camel and buffalo

**DOI:** 10.14202/vetworld.2020.354-359

**Published:** 2020-02-26

**Authors:** Ali F. Bargooth, Jafar G. A. Ali-Jebori, Ahmed M. Al-Badri, Ali M. R. Al-Yasari, Esraa A. Zegyer

**Affiliations:** 1 Department of Biology, College of Education for Pure Sciences, Wasit University, Iraq; 2 Department of Anatomy, College of Veterinary Medicine, Qasim Green University, Iraq; 3 Department of Biology, College of Science, Wasit University, Iraq; 4 College of Veterinary Medicine, Al-Muthanna University, Iraq

**Keywords:** buffalo, camel, glucagon, immunohistochemical, insulin, pancreas

## Abstract

**Background and Aim:**

Glucagon plays a significant role in glucose homeostasis by controlling hepatic glucose output in terms of both hypoglycemic and normoglycemic conditions. This study aimed to determine the amount and intensity of insulin and glucagon in addition to estimating the relationship between α- and β-cells for two animals, camel and buffalo.

**Materials and Methods:**

Twenty fresh pancreas samples were collected from 10 buffalo and 10 camel adults immediately after slaughter from AL-Kut abattoir, Al- Kut, Iraq. Hematoxylin and eosin staining technique and the immunohistochemistry technique were used.

**Results:**

The histological results, for both animals, showed the cells of the pancreatic islet could be differentiated from the exocrine cells by their paler appearance. The pancreatic islets were round, oval, and irregular shaped. In the camel, the pancreatic islets had a larger diameter than that in the buffalo. The average diameter of β-cells and their percentage was higher than those of the α-cells in the camel. In the buffalo, glucagon-immunoreactive cells were found in abundance with high intensity, whereas insulin-immunoreactive cells were more prominent with high intensity in the camel. In both animals, the α-cells and glucagon-immunoreactive cells were distributed on the peripheries of the pancreatic islets, whereas the β-cells were distributed throughout the pancreatic islets.

**Conclusion:**

The study inferences that these differences may be due to the differences in the environment of the animals which affect the structures of body organs.

## Introduction

Buffalo and camel are two types of cattle in Iraq. Both are used to produce meat and milk and are consumed daily in Iraq. The Indian buffalo, also known as the water buffalo, is the most common type of buffalo in Iraq; having been introduced from India about 13 centuries ago, it has adapted considerably to the prevailing conditions of the country [1,2]. The water buffaloes are acclimatized to live in a moist tropical climate, especially in places that support rich, permanent pastureland and are regarded as a poor thermoregulating animal due to many reasons, namely, weakness of the sweat gland, dark discoloration of the skin, the presence of a great amount of fat in the tissue, and small surface area/volume ratio [3,4]. *Camelus* is the common type of camel in Iraq. It comprises two species. The first is the dromedary camel or the one-humped camel (*Camelus dromedarius*) defined as the Arabic camel that has spread in the hot deserts of Africa and the deserts of Asia. The second is the Bactrian camel or the two-humped camel (*Camelus bactrianus*) that is distributed in the cold desert and dry plains of Asia [5]. The well-adapted anatomical, histological, and physiological features of the camel help it survive tough desert conditions [6]. Adaptations (phenotype change) are the result of evolution. Evolution (genotype) is a change in a species over extended periods of time. The adaptations in desert animals are aimed at conserving water and regulating body temperature. The desert animals are confronted with harsh desert conditions such as scarcity of water and nutrition, sand and stone surfaces, desert plants, and the hot and dry weather [7].

The mammalian pancreas is a compound tubuloacinar gland with exocrine and endocrine parts. Some digestive enzymes are secreted from the exocrine and stored within. The endocrine consists of aggregates of different cells, defined as the islets of Langerhans that are distributed among the acini [8]. The endocrine cells of the mammalian pancreas are represented primarily by α-cells (tumor necrosis factor-α or glucagon-producing cells), β-cells (insulin-producing cells), Δ-cells (somatostatin-producing cells), and PP cells (F-cells, pancreatic polypeptide-producing cells) [9]. The function of each of the endocrine secretions and interactions is of particular importance to the natural functioning of the body. Endocrine cells produce hormones that are indispensable, such as glucose-dependent insulinotropic peptide and glucagon which are considered critical to the optimal performance of body metabolism [10]. The glucagon hormone is considered a peptide hormone and is secreted by the α-cells. Glucagon induces high concentrations of glucose in the bloodstream, whereas the insulin hormone has the opposite effect, which is the depression of glucose concentrations [8]. The α-cells that co-occupy the islets of Langerhans in association with β-cells have long been recognized as the glucagon source, a diabetogenic and hyperglycemia-producing hormone. However, the mechanisms that control the functions of α-cells, secretion of glucagon, and the role of glucagon in diabetes have remained somewhat enigmatic during the 50 years since they were discovered. Seminal findings throughout the past several years have brought α-cells into the focus of scientific discovery. The findings obtained mainly from studies in mice are as follows: α-cells have the capability to transdifferentiate into insulin-producing β-cells and that α-cells have a glucagon-like peptide-1 (GLP1)-producing system that creates GLP1 locally for paracrine activities inside the islets which usually likely promotes the growth of β-cells and maintains and ensures the survival of the β-cell mass. Impairment of glucagon signaling helps to both prevent the occurrence of diabetes in conditions of near absence of insulin and increases the mass of α-cells. The α-cells seem to work as assistant cells or guardians of β-cells ensuring their health and well-being. The observation of the impairment of glucagon signaling which results from probably due to promoting the transformation of α-cells to β-cells, which causes a noticeable rise in the mass of α-cells in the islets. Such α-cells hyperplasia provides an increased supply of α-cells for their transdifferentiation into new β-cells [11].

There is a paucity of immunohistochemical studies regarding the insulin and glucagon hormones, especially the comparison between different animal species. Therefore, this study was designed to determine the immunohistochemical features of these hormones in relation to differences in the environment of the camel and buffalo.

## Materials and Methods

### Ethical approval

This research was worked with sponsorship and approval from Wasit University Research Bureau, Iraq, and the agreement of the College of Education for Pure Sciences, Iraq.

### Histological technique

Twenty fresh pancreas samples (10 each from adult buffalos and camels) were collected immediately after slaughter from AL-Kut abattoir, Al-Kut, Iraq. All animals were clinically healthy. After the pancreas samples were isolated, they were fixed in 10% formalin for 72 h and washed in tap water for 2-3 h. Then, the samples were subjected to numerous histological techniques as follows: Dehydration, clearing, infiltration, embedding, cutting, and staining with hematoxylin and eosin (H&E) stain for displaying the general structure of the tissue and staining with aldehyde fuchsin stain to display and distinguish the pancreatic islet cells [12].

### Histomorphometrical study

Parameters such as the diameter of the islets of Langerhans, α-cells, and β-cells and the percentages of α-cells and β-cells in the islets of Langerhans were measured using ocular micrometers. The average of these measurements was computed and used for analysis. Data were subjected to the t-test at a significance level of p≤0.05 using SPSS [13].

### Immunohistochemistry technique

The immunohistochemistry process was performed using a mouse or rabbit polyclonal antibody against insulin and glucagon. This process was used to detect the insulin and glucagon hormones in the pancreas. Briefly, sections from embedded pancreas were dewaxed, hydrated in a decreasing series of graded alcohol solutions, and then washed thrice in distilled water. Antigen retrieval was performed by immersing the slides in a jar containing a citrate buffer solution (pH 6). Slides were washed with phosphate buffer saline (PBS; pH 7.2) for 5 min. Sections were incubated with peroxidase block reagent for 5-10 min at room temperature (37°C) washed thrice with distilled water, and then with PBS. Subsequently, sections were incubated with protein blocking solution for 5-10 min at (37°C) and then incubated with primary antibody (diluted at 1:500) for 30 min at (37°C). Slides were washed with PBS 5-7 times and then incubated with one-step horseradish peroxidase polymer for 30 min at (37°C). Then, slides were first washed with PBS and later with distilled water 2-3 times. A few drops of ready-to-use 3,3’-Diaminobenzidine reagent was added to the tissue slides (1 mL of buffer reagent and substrate mixed well and 50 µL of DAB chromogen (C reagent) for 6-10 min at (37°C), and then, slides were first washed with PBS and later in distilled water. Subsequently, the sections were incubated with H&E stain for 30-60 s. Then, slides were washed with distilled water and mounted using dibutylphthalate polystyrene xylene mounting medium. The intensity of glucagon and insulin was measured using Image J and statistical analysis for data of intensity was performed using the t-test at a significance level of p≤0.01 using GraphPad Prism.

## Results

This study describes the main differences in the characteristics of the pancreatic islets in the camel and buffalo such as the size of the islets along with the description and observations regarding the distribution and amount of β-cells and α-cells.

**Figure-1:**
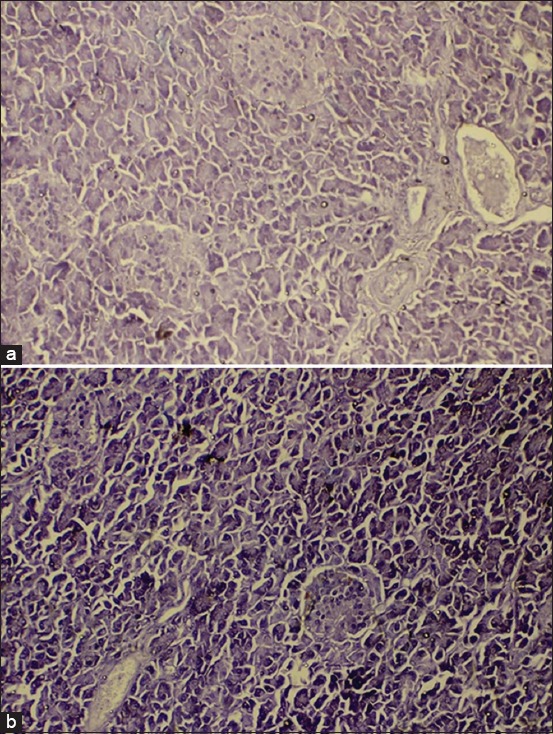
Photomicroscope of the pancreas of the camel (a) and buffalo (b), in both animal pancreatic islets had oval, round, and irregular shape (arrows). Hematoxylin and Eosin, 100×.

The pancreatic endocrine was arranged in a pancreatic gland in both animals (camel and buffalo) as a mass with round, oval, and irregular shapes; this mass of cells is called the pancreatic islets or islets of Langerhans. The pancreatic islet cells can be differentiated from the exocrine cells due to their paler appearance. The boundary of the connective tissue was very thin; it surrounded the pancreatic islets and separated the islets from the exocrine units. The capillaries surrounded the islets providing contact with the endocrine cells in the islets (Figure-1).

**Figure-2:**
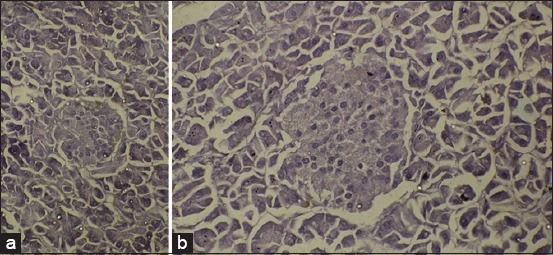
Photomicroscope of the pancreas of the camel (a) and buffalo (b), alpha-cell (yellow arrows), beta-cell (white arrows), and oval-shaped nucleus were referred to α-cell and round-shaped nucleus were referred to the β-cells. Hematoxylin and Eosin, 400×.

The β-cells and α-cells could be distinguished and identified based on the shapes of the nucleus. The cells with an oval-shaped nucleus were referred to as α-cells and those with a round-shaped nucleus were referred to as the β-cells (Figure-2).

The average diameter of the pancreatic islets in the camel was substantially higher than that in the buffalo. The average diameter of the α-cells in the buffalo recorded significantly higher values than that in the camel, whereas the average diameter of β-cells in the camel recorded higher values than that in the buffalo (Table-1).

**Table-1:** Measurements the diameters of the pancreatic islets, diameters of α-cells, and diameters of β-cells camel and buffalo.

Animals	The diameter of pancreatic islets/μm (Mean±SD)	The diameter of α-cells/μm (Mean±SD)	The diameter of β-cells/μm (Mean±SD)
Camel	98.03±0.51^a^	8.90±0.18^a^	8.45±0.11^a^
Buffalo	88.22±0.44^b^	10.01±0.16^b^	7.92±0.35^b^

*Different small letters (a, b) are significant (p≤0.05) to compression rows

The distribution of β-cells in the camel was 77-88%, higher than that in the buffalo at 63-69%. In contrast, α-cells comprised 10-17% and 25-27% in the camel and buffalo, respectively.

**Figure-3:**
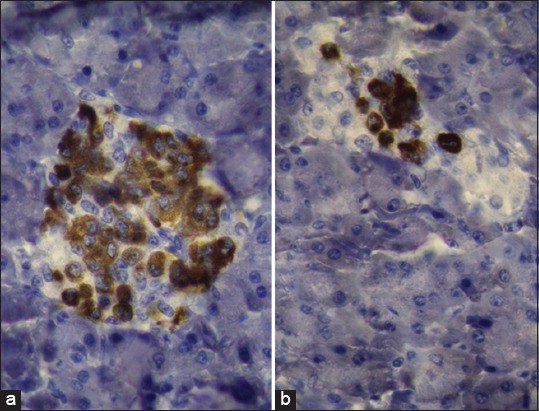
Photomicroscope of the pancreas of the camel (a) and buffalo (b) using immunostaining of insulin hormone-producing cells. In both animals, beta-cells were spread in different areas (peripheral center) of islets (red color), but the amount of the insulin hormone in camel had higher than in buffalo, 400×.

**Figure-4:**
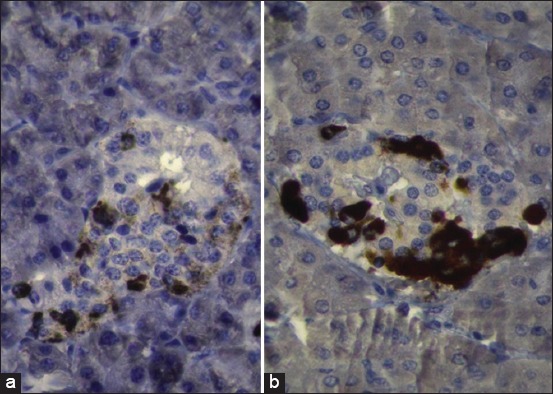
Photomicroscope of the pancreas of the camel (a) and buffalo (b) using immunostaining of glucagon hormone-producing cells. In both animals, alpha-cells were spread in peripheral areas of islets (red color), but the amount of the glucagon hormone in buffalo had higher than in camel, 400×.

**Figure-5:**
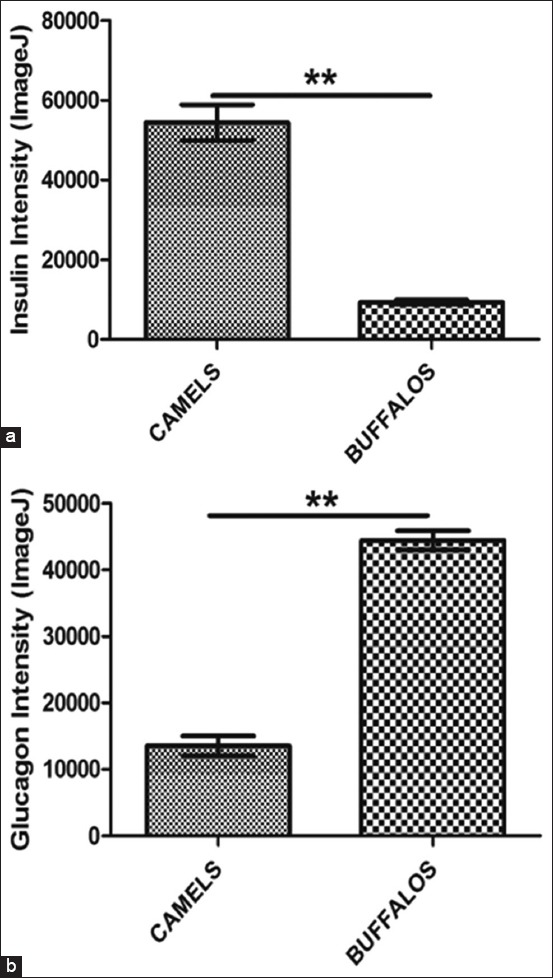
The intensity of insulin hormone (a) and glucagon hormone (b) in the camels and buffalo. **The significant differences at p≤0.01 to compression column, using Image J program and the statistical analysis of t-test at significant differences limited on p≤0.01 of probability was done for data of intensity using GraphPad Prism program. The intensity of insulin hormone (a) of camel had higher than in buffalo, while the intensity of glucagon hormone (b) of buffalo had higher than in camel.

The immunohistochemical study showed the spread of β-cells in different areas of the islets in both animals (camel and buffalo). The amount and intensity of the glucagon hormone in the pancreatic islets of the buffalo were higher than that in the pancreatic islets of the camel. In contrast, the insulin hormone was higher in amount and intensity in the camel than in the buffalo. Distribution of the α-cells was at the periphery of the pancreatic islets in both animals; the amount of insulin hormone was higher in the pancreatic islets of the camel than that of the buffalo. Both α- and β-cells in the camel and buffalo were observed outside the pancreatic islets in the connective tissue (Figures3-5).

## Discussion

In the present study, the pancreatic endocrine within the pancreatic gland was observed to be a mass of cells with round, oval, and irregular shapes in both the camel and buffalo. The present observation is similar to that of Zghair [14] who reported that the shape of islets in the camel was round or oval and showed that the islets of the pancreas had different shapes in the camel. The differences in shape in the islets of the pancreas may be due to the transport of endocrine cells from the islets to the exocrine tissue or because no strong capsule was observed to surround the islets.

The previous studies have reported variations in the size of the pancreatic islets in different animals as in the camel [15], the Mizo local pig [16], and the sheep and dog [17]. The different sizes of the pancreatic islets may be related to the different requirements of secretions of these islets for metabolism in each animal, which depends mainly on the glucose level in the blood and affects the cell types and size of islets [18].

The identification of cells with the oval-shaped nucleus as α-cells and those with the round-shaped nucleus as the β-cells is similar to the description given by Zghair [14] in the camel. The results of the previous studies are both consistent and contradictory to the results of our study regarding the spread of α-cells in the periphery of the islets and β-cells in different areas of the islets. A similar description has been provided for the camel [14], whereas our results are different from those reported by Hafez *et al*. [19] that the β-cells were spread in the periphery of the islets and the α-cells were spread centrally in the horse. In humans, the endocrine cells are intermingled [20]. Distribution and juxtaposition of the α-cells and β-cell in pancreatic islets effect on the functions of these cells, through the cell-cell interactions. The secretion of hormones (glucagon and insulin) has an effect on these interactions through positive-negative insulin feedback according to the need for these hormones in different species. The different distribution of these cells is related to their regulatory mechanism [21]. The increase in the size of the pancreatic islets, the increase in the percentage and diameter of β-cells, and high amount of insulin hormones with a decrease in the percentage and diameter of α-cells, and low amount of glucagon hormone in camels compared with that in the buffalo are related to the structures and functions of the organs in the body. Hussin [22] reported that the different environments of animal species affect the structures of organs as in the acclimatization of the camel to a harsh terrestrial environment; therefore, it has long loop nephrons in the kidney to reabsorb a larger amount of water and substances to maintain normal thermoregulation. Alkinanni [23] explained that the buffalo has short loop nephrons to excrete more water along with urine to balance thermoregulation because it has thick skin with poor sweat glands, so there is an increase in blood sugar and related components. Gerich [24] reported that the kidney can enable the gluconeogenesis process by producing new molecules of glucose and releasing these for circulation. The kidney can also affect glucose homeostasis through returning glucose into circulation through the reabsorption of glucose from the glomerular filtrate. Ca^++^ and K^+^ ions are important in mediating the release of the insulin hormone by the β-cells, as noted by Hussin [22]. In addition, Rutter [20] showed the capacity of the camel to tolerate thirst and lack of water by controlling the levels of K^+^ and Ca^++^ ions that affect the release of insulin. Based on the reabsorption of glucose as described above, we can conclude that the camel likely needs a high amount of insulin to maintain the level of glucose, unlike the buffalo which needs a high amount of glucagon to raise the level of blood glucose in the blood.

## Conclusion

The results of our study regarding the spread of α-cells in the periphery and that of β-cells in different areas of the pancreatic islets in both animals were both consistent and contradictory to the previous observations. The presence of larger pancreatic islets, larger and more numerous β-cells, and a high amount of insulin hormone and smaller and fewer α-cells and a low amount of glucagon hormone in camels in comparison to that observed in the buffalo are related to the structures and functions of the organs in the body. A likely reason is that the need for maintenance of glucose levels in the blood results in the requirement for high amount of insulin in the camel as opposed to requiring a raised level of blood glucose resulting in the need for a high amount of glucagon in the buffalo.

## Authors’ Contributions

Conceptualization and formal analysis, AFB; funding acquisition and investigation, JGAA; methodology, project administration, and resources, AMA; software, supervision, and validation, AMRA; writing – original draft, writing – review, and editing, EAZ. All authors read and approved the final manuscript.
